# Generation and Characterization of a Nanobody Against SARS-CoV

**DOI:** 10.1007/s12250-021-00436-1

**Published:** 2021-08-17

**Authors:** Jiang-Fan Li, Lei He, Yong-Qiang Deng, Shu-Hui Qi, Yue-Hong Chen, Xiao-Lu Zhang, Shi-Xiong Hu, Rui-Wen Fan, Guang-Yu Zhao, Cheng-Feng Qin

**Affiliations:** 1 State Key Laboratory of Pathogen and Biosecurity, Beijing Institute of Microbiology and Epidemiology, Academy of Military Medical Sciences (AMMS), Beijing, 100071 China; 2 College of Veterinary Medicine, Shanxi Agriculture University, Jinzhong, 030801 China; 3 Research Unit of Discovery and Tracing of Natural Focus Diseases, Chinese Academy of Medical Sciences, Beijing, 100071 China

**Keywords:** Severe acute respiratory syndrome coronavirus (SARS-CoV), Receptor-binding domain (RBD), Nanobody, Neutralizing antibody

## Abstract

The sudden emergence of severe acute respiratory syndrome coronavirus (SARS-CoV) has caused global panic in 2003, and the risk of SARS-CoV outbreak still exists. However, no specific antiviral drug or vaccine is available; thus, the development of therapeutic antibodies against SARS-CoV is needed. In this study, a nanobody phage-displayed library was constructed from peripheral blood mononuclear cells of alpacas immunized with the recombinant receptor-binding domain (RBD) of SARS-CoV. Four positive clones were selected after four rounds of bio-panning and subjected to recombinant expression in *E. coli*. Further biological identification demonstrated that one of the nanobodies, S14, showed high affinity to SARS-CoV RBD and potent neutralization activity at the picomole level against SARS-CoV pseudovirus. A competitive inhibition assay showed that S14 blocked the binding of SARS-CoV RBD to either soluble or cell-expressed angiotensin-converting enzyme 2 (ACE2). In summary, we developed a novel nanobody targeting SARS-CoV RBD, which might be useful for the development of therapeutics against SARS.

## Introduction

Coronaviruses are a large family of viruses within the family *Coronaviridae*, and the order *Nidovirales*, which are mainly divided into four groups. They may cause diseases of different severities in various animals (Zaki *et al*. 2012). The first human coronavirus that caused a global outbreak was severe acute respiratory syndrome coronavirus (SARS-CoV), which was first found in Guangdong Province, China in November 2002 (Ksiazek *et al.* 2003; Peiris *et al*. 2003). Several weeks later, it had spread to 25 countries causing at least 8000 infections. By July 3, 2003, 8439 cases had been reported, resulting in 812 deaths (Kuiken *et al*. 2003). On July 5, 2003, the World Health Organization declared the world free of ongoing SARS transmission. However, there were four sporadic SARS cases reintroduced from animals in Guangdong China in late 2003 and early 2004, of which the virus isolates were different from the previous outbreak (Liang *et al.* 2004). In total, three instances of laboratory-acquired infections occurred in Singapore, Taiwan and Beijing (Anderson and Tong 2010). Thus, the possibility of reintroduction of SARS-like-CoVs from animals and the leakage of SARS-CoV from the laboratory are still concerned (Du *et al*. 2009). Although no case has been reported for years, there is a possibility of a new outbreak of SARS. Unfortunately, there are no effective antivirals or licensed vaccines to treat or prevent SARS.

As they both belong to lineage B of the *Betacoronavirus* genus, SARS-CoV, and the ongoing disastrous SARS-CoV-2 share 79% genome sequence identity (Lu *et al*. 2020), the latter of which has led to over 194 million infections and 4 million deaths (https://covid19.who.int). The genome of SARS-CoV encodes four major structural proteins: spike (S), membrane (M), envelope (E), and nucleocapsid (N) proteins (Tsunetsugu-Yokota *et al*. 2006). The S protein is a type I membrane glycoprotein that consists of two distinct functional domains. The S1 domain near the amino terminus mediates the binding between SARS-CoV and its receptor, angiotensin-converting enzyme 2 (ACE2). The S2 domain near the carboxy terminus mediates membrane fusion. Neutralizing antibodies are mainly induced by the S protein (Gallagher and Buchmeier 2001). Furthermore, the receptor-binding domain (RBD), which is located in the middle of S1 where S interacts with ACE2, contains neutralizing epitopes that induce potent neutralizing antibodies in animal models (He *et al*. 2004).

Neutralizing antibodies play an important role in protecting against infectious diseases. Previous studies have reported that SARS patients receiving SARS convalescent plasma therapy showed clinical improvement (Pearson *et al*. 2003; Cheng *et al*. 2005). Moreover, passive transfer of serum from mice immunized with SARS-CoV to naïve mice resulted in reduced lung viral load following virus challenge (Subbarao *et al*. 2004). A monoclonal antibody-based on the B cells of a convalescent patient significantly reduced the viral load in the upper respiratory tract of mice that received antibody treatment before challenge (Traggiai *et al*. 2004). Another monoclonal antibody identified from immunized human immunoglobulin transgenic mice alleviated viral load and associated pathological findings in a golden Syrian hamster model after exposure (Roberts *et al*. 2006). These results revealed that neutralizing antibodies are potent prophylactic and therapeutic agents.

Nanobodies from heavy chain antibodies naturally occur in camels (Hamers-Casterman *et al*. 1993), representing an attractive weapon against infectious diseases. Its size, 2.5 nm in diameter and nearly 4 nm high, with a molecular weight of about 15 kDa, makes it the smallest antigen-binding antibody fragment to date (Wang *et al*. 2019). It is quite stable at extreme temperatures and pH values (Ebrahimizadeh *et al*. 2013). It can also recognize unique epitopes that are not accessible to conventional antibodies (Lauwereys *et al*. 1998). These characteristics give nanobodies their great therapeutic value.

In this study, we established a phage-displayed nanobody library from alpacas immunized with recombinant SARS-CoV RBD protein. Further screening and characterization identified a nanobody, S14, with potent neutralization against SARS-CoV. The platform described here provides a useful tool to rationally develop novel nanobodies against emerging viruses with global impact.

## Materials and Methods

### Cells and Proteins

All cells used in this study were cultured at 37 °C with 5% CO_2_. Dulbecco’s minimum essential medium (DMEM) complete medium containing 10% fetal bovine serum and 1% penicillin/streptomycin was used for cell growth and replaced with DMEM without any additives after transfection. 293T cells were used for recombinant RBD protein expression and SARS-CoV pseudovirus production. HeLa cells stably expressing human ACE2 (HeLa-ACE2), kindly provided by Prof. Zheng-Li Shi (Wuhan Institute of Virology, Chinese Academy of Sciences), were used for flow cytometry.

Recombinant SARS-CoV RBD protein fused with rabbit IgG-Fc tag (RBD-rFc; SinoBiological) was used for alpacas immunization, bio-panning, identification, and flow cytometry. Recombinant rabbit IgG-Fc protein (rFc; SinoBiological) was used to remove rFc-specific phage antibodies during bio-panning.

### Alpacas Immunization

A one-year-old female alpacas was subcutaneously immunized with 200 μg recombinant SARS-CoV RBD-rFc protein plus Freund’s complete adjuvant, and boosted three times with the same protein plus Freund’s incomplete adjuvant every two weeks. Before immunization, blood samples of alpacas were collected for SARS-CoV-RBD-specific antibody detection. The housing and care of alpacas and the study protocols were approved by the Animal Experimentation Ethics Committee of Shanxi Agricultural University (2018).

### Construction of SARS-CoV-RBD-Specific Phage-Displayed Nanobody Library and Bio-Panning

**Fig. 1:**
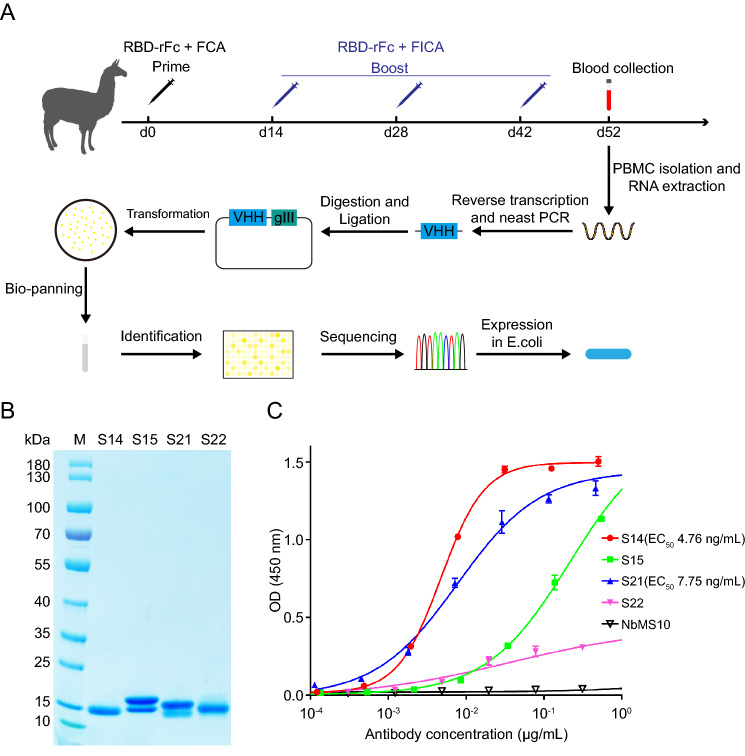
Generation and identification of SARS-CoV-RBD-specific nanobody. **A** Schematic illustration of the construction of SARS-CoV nanobody library and generation of SARS-CoV-RBD-specific nanobody. *RBD-rFc* SARS-CoV RBD with rabbit IgG-Fc tag (Sino biological), *FCA* Freund's complete adjuvant, *FICA* Freund's incomplete adjuvant, *VHH* variable region of heavy chain antibody. **B** SDS-PAGE analysis of purified SARS-CoV nanobodies. *M* Marker. The molecular weight is indicated on the left. **C** Evaluation of the binding activity between SARS-CoV RBD and nanobodies. Results are presented as the mean values of optical density at the absorbance of 450 nm ± standard deviation (n = 2).

A phage-displayed library was constructed as previously described (Zhao *et al*. 2018). Briefly, ten days after the final immunization, anticoagulant blood was collected from the jugular vein (Fig. 1). Peripheral blood mononuclear cells (PBMCs) were isolated by Ficoll-Paque gradient centrifugation and total RNA was extracted using TRIzol reagent. First-strand cDNA synthesis was performed by reverse transcription PCR using total RNA as a template. The gene of the variable region of the heavy chain of heavy chain antibody (VHH) was amplified by nested PCR. The first primer pair (forward: 5′-GGTGGTCCTGGCTGC-3′; reverse: 5′-GGTACGTGCTGTTGAACTGTTCC-3′) amplified two segments. The smaller band (~ 700 bp) was retrieved to amplify the VHH gene using the second primer pair (forward: 5′-TTTCTATTACTAGGCCCAGCCGGCCGAGTCTGGAGGRRGCTTGGTGCA-3′; reverse: 5′-AAACCGTTGGCCATAATGGCCTGAGGAGACGRTGACSTSGGTC-3′) (the *Sfi*I restriction site is underlined). The *Sfi*I-digested VHH and phagemid vector pCANTAB5E were ligated using T4 ligase (Thermofisher) and electro-transformed into TG1. SARS-CoV RBD-rFc protein and rabbit IgG-Fc protein were used for bio-panning. After four rounds of panning, monoclonal phages were identified using ELISA. Four positive clones were selected for expression.

### Recombinant Protein Expression of SARS-CoV-RBD-Specific Nanobodies and SARS-CoV RBD with Its Mutants

The *VHH* genes of the four positive clones were cloned into the protokaryon vector pCold I (Takara) and transformed into *E.coli*. The recombinant nanobodies with an N-terminal 6 × His tag were expressed in *E.coli* induced by isopropyl-beta-D-thiogalactopyranoside (IPTG) and purified using Ni–NTA resin.

The recombinant SARS-CoV RBD and its mutant proteins (Y442A, L472A, N479A, D480A, and T487A) fused with human IgG-Fc tag (SinoBiological) were expressed in 293T cells using the eukaryotic vector pFUSE-IgG1-Fc2 and purified by Protein A resin.

### ELISA

The binding activity between SARS-CoV RBD and nanobodies was detected by ELISA. The ELISA plate was coated with SARS-CoV RBD-rFc protein (or mutant RBD-Fc proteins for epitope analysis) at 1 μg/mL overnight at 4 °C. After blocking with 3% BSA for 1 h at 37 °C, serially diluted nanobodies were added and incubated for 45 min at 37 °C. Next, horseradish peroxidase (HRP)-conjugated mouse anti-His-tag antibody (1:5000) was added and incubated for 30 min at 37 °C. And then 3,3′,5,5′-tetramethylbenzidine (TMB) substrate was added and stopped with 1 N H_2_SO_4_. The absorbance at 450 nm was measured using a microplate reader (BioTeck). A MERS-CoV-RBD-specific nanobody NbMS10 (Zhao *et al*. 2018) was used as a negative control.

Competitive ELISA was performed in a similar manner. Briefly, the plate was coated with SARS-CoV S1 protein (SinoBiological) at 6 μg/mL overnight at 4 °C. After blocking with 3% BSA for 1 h at 37 °C, ACE2 protein fused with human IgG-Fc tag (SinoBiological) at 6 μg/mL and serially diluted nanobodies were simultaneously added and incubated for 45 min at 37 °C. Then, HRP-conjugated goat anti-Fc-tag antibody (1:4000) was added and incubated for 30 min at 37 °C. The subsequent procedures were the same as described above.

### Pseudovirus Neutralization Assay

The SARS-CoV pseudovirus neutralization assay was performed as previously described (Zhao *et al*. 2013). Briefly, 293T cells were co-transfected with a plasmid encoding Env-defective and luciferase-expressing HIV-1 genome (pNL4-3.Luc.R-.E-) and a plasmid expressing SARS-CoV spike protein. After 36 h, the supernatant containing SARS-CoV pseudovirus particles was harvested and stored at − 80 °C. Then, 500 TCID_50_ of SARS-CoV pseudovirus was incubated with serially diluted nanobodies for 1 h at 37 °C. Huh-7 cells were added and cultured for 48 h. Substrate (PerkinElmer) containing lysis was added and reacted for 2 min. The mixture was transferred into a white plate and the relative light unit (RLU) was read using a luminometer (Promega). The inhibition rate was calculated as follows:

$$ {\text{Inhibition rate }}\left( \% \right) \, = \, \left[ {\left( {\text{RLU of pseudovirus control}} \right) - \left( {\text{RLU of samples}} \right)} \right]/\left[ {\left( {\text{RLU of pseudovirus control}} \right) - \left( {\text{RLU of cell control}} \right)} \right] \times {1}00\% . $$

The IC_50_ (the 50% inhibition concentration) was calculated by GraphPad Prism.

### Bio-layer Interferometry

The binding affinity between SARS-CoV RBD-Fc and S14 was measured using a gator (Probe Life). Recombinant SARS-CoV RBD-Fc protein (100 nmol/L) was captured using anti-Fc probes. The probes were individually inserted into buffer (0.2% IgG-free BSA and 0.01% Tween20 in PBS) containing S14 of different concentrations (from 25 nmol/L to 1.56 nmol/L) for association and then inserted into buffer (0.2% IgG-free BSA and 0.01% tween20 in PBS) for disassociation. The wave shifts were analyzed using Gator software and fitted to a 1:1 binding model.

### Flow Cytometry

Serially diluted S14 or PBS was incubated with SARS-CoV RBD-rFc (rabbit Fc) protein (5 μg/mL) for 30 min at room temperature. Hela-ACE2 cells were then added and incubated for another 30 min at 37 °C. Fluorescein isothiocyanate (FITC)-conjugated goat anti-rabbit antibody (1:100) was added and incubated for 30 min at 37 °C. The cells were analyzed via flow cytometry. NbMS10 was used as the negative control.

## Results

### Generation of SARS-CoV-RBD-Specific Nanobodies

SARS-CoV-RBD-specific nanobodies were generated using standard phage display technology from alpacas immunized with recombinant SARS-CoV RBD-rFc (Fig. 1A). After four rounds of panning and phage clone identification, a total of four positive clones, named S14, S15, S21 and S22, were selected by ELISA. Then, their *VHH* genes were subcloned into an *E. coli* expression vector, and nanobodies were purified via Ni–NTA affinity chromatography and verified by SDS-PAGE. The results showed that the molecular weight of the four nanobodies was approximately 16 kDa, which was in agreement with their theoretical molecular weights (Fig. 1B). ELISA results further showed that three of the four nanobodies (S14, S15, and S21) bound well and S22 bound weakly to recombinant SARS-CoV RBD-rFc, while the MERS-CoV nanobody NbMS10 showed no binding activity (Fig. 1C).

### Characterization of SARS-CoV Nanobody S14

**Fig. 2:**
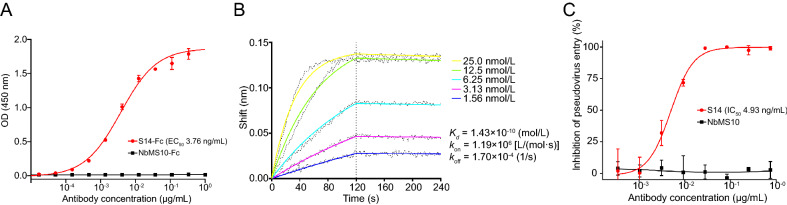
Characterization of SARS-CoV nanobody S14. **A** Evaluation of the binding activity between SARS-CoV S and nanobody S14. Results are presented as the mean values of optical density at the absorbance of 450 nm ± standard deviation (n = 2). **B** Bio-layer interferometry. The light-wave shifts were recorded and curves were fitted by gator (Probe life). **C** SARS-CoV pseudovirus neutralization assay. The results are presented as the mean inhibition rates ± standard deviation (n = 2).

**Fig. 3:**
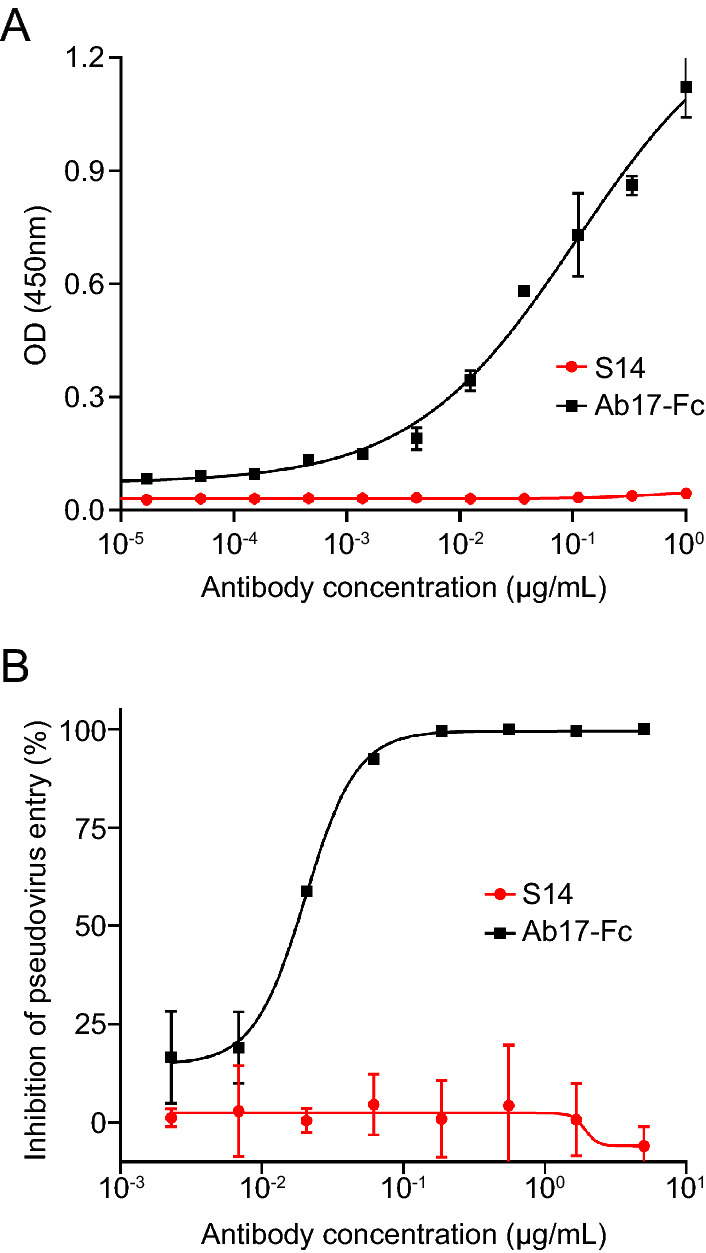
Evaluation of the cross-reaction of nanobody S14 against SARS-CoV-2. **A** Evaluation of the binding activity between SARS-CoV-2 RBD and nanobody S14. Results are presented as the mean values of optical density at the absorbance of 450 nm ± standard deviation (n = 2). **B** SARS-CoV-2 pseudovirus neutralization. The results are presented as the mean inhibition rates ± standard deviation (n = 2). Ab17-Fc: A monoclonal antibody against SARS-CoV-2.

Based on the ELISA results, S14 was chosen for further characterization. The results of another ELISA showed that S14 fused with human IgG-Fc (S14-Fc) bound well to recombinant SARS-CoV S with an EC_50_ of 3.76 ng/mL, while the MERS-CoV antibody NbMS10-Fc showed no binding activity (Fig. 2A). Bio-layer interferometry was performed to determine the binding kinetics of S14 to SARS-CoV RBD. The results showed that S14 has a favorable binding activity to SARS-CoV RBD, with an equilibrium dissociation constant (*K*_d_) of 143 pmol/L (Fig. 2B). The results of the SARS-CoV pseudovirus neutralization assay showed that S14 could efficiently inhibit the entry of pseudovirus into the target Huh-7 cells, with an IC_50_ of 4.93 ng/mL (Fig. 2C), while NbMS10 showed no inhibitory activity, as expected. Meanwhile, S14 showed no binding activity against SARS-CoV-2 RBD (Fig. 3A) nor neutralization activity against SARS-CoV-2 pseudovirus (Fig. 3B). These results demonstrate that S14 is a potent SARS-CoV-specific neutralizing nanobody.

### Neutralization Mechanism of SARS-CoV Nanobody S14

**Fig. 4:**
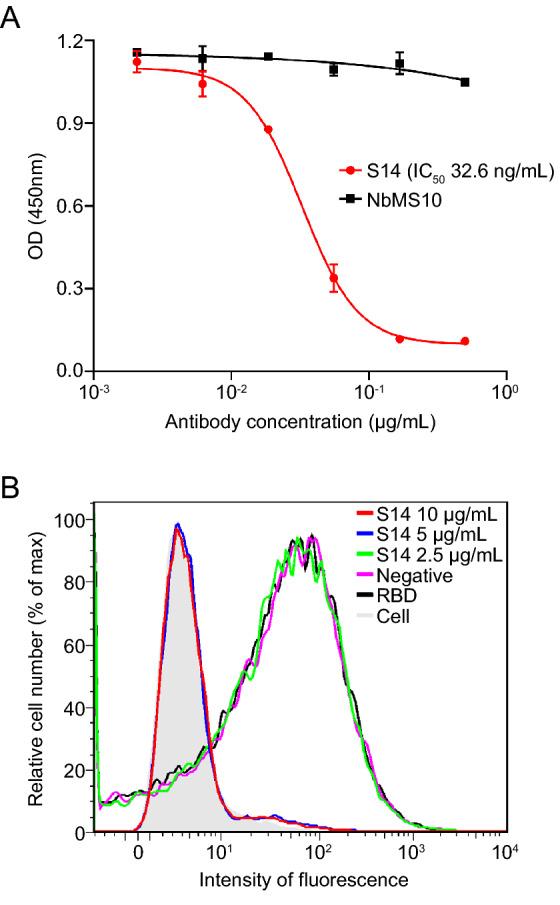
Neutralization mechanism of SARS-CoV nanobody S14. **A** Competitive ELISA. Results are presented as the mean values of optical density at the absorbance of 450 nm ± standard deviation (n = 2). **B** Flow cytometry analysis of S14 in inhibiting the binding between SARS-CoV RBD and cell-associated ACE2 receptor.

To investigate the mechanism of S14 mediated neutralization, competitive binding assays were performed. As shown in Fig. 4A, S14 blocked the binding between soluble ACE2 and SARS-CoV S1 in a dose-dependent manner, while the negative control NbMS10 failed to do so. Meanwhile, the flow cytometry assay also showed that high concentrations of S14 blocked the binding of SARS-CoV RBD to recombinant ACE2 expressed in Hela-ACE2 cells. However, a low concentration of S14 and NbMS10 did not affect RBD-ACE2 binding (Fig. 4B). These results showed that S14 directly impaired the interaction between SARS-CoV RBD and ACE2.

### Epitope Analysis

**Fig. 5:**
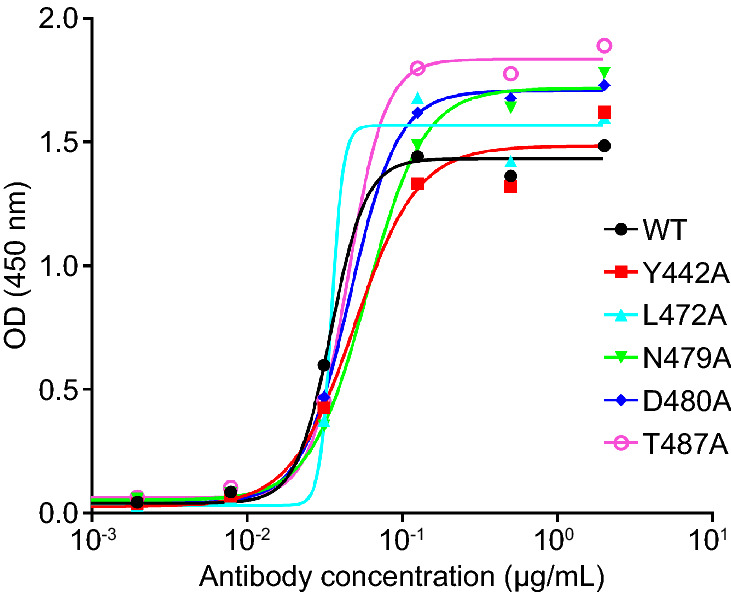
Epitope analysis of nanobody S14 binding to SARS-CoV RBD. The results are presented as values of optical density at the absorbance of 450 nm.

The structure of SARS-CoV spike RBD complexed with ACE2 has already been well resolved (Li *et al*. 2005). In the complex interface, residues Y442, L472, N479, D480, and T487 play an important role in host tropism and cross-species infections of SARS-CoV (Li 2013). To determine whether these mutations were involved in S14-mediated neutralization, we produced a series of RBD mutants, including Y442A, L472A, N479A, D480A, and T487A, and subjected them to ELISA. The results showed that S14 reacted well with all of the above mutants (Fig. 5), suggesting that the neutralization activity of S14 was not affected by these specific mutations.

## Discussion

In this study, using routine phage display technology, we identified a nanobody S14 with high binding affinity to SARS-CoV RBD (*K*_d_ = 143 pmol/L) as well as inhibition of pseudovirus entry with an IC_50_ of 4.93 ng/mL, which was expected to be a potent candidate. SARS-CoV RBD is a major determinant (Chen *et al*. 2005). Several antibodies targeting RBD have shown promising therapeutic value *in vitro* and *in vivo*. MAb201 (Greenough *et al*. 2005), generated from human immunoglobulin transgenic mice, showed an affinity constant (*K*_d_) of 34 nmol/L with S590 (aa 1–590). This could significantly decrease the viral load in mice challenged with SARS-CoV. The human IgG1 form of 80R (Sui *et al*. 2004), generated from a non-immune human antibody library, had a comparable binding affinity (*K*_d_ = 1.59 nmol/L) to S1 with the receptor ACE2 (*K*_d_ = 1.70 nmol/L). It reduced more than 4 logs of lung viral load in BALB/c model at 12.5 mg/kg of body weight (Sui *et al*. 2005). Another antibody, IgG1 m396 (Prabakaran *et al*. 2006), with the best binding affinity (*K*_d_ = 4.6 pmol/L) has been reported to inhibit pseudovirus entry with an IC_50_ of 10 ng/mL. Considering the high correlation between pseudovirus and authentic coronavirus (Han *et al*. 2004; Zhao *et al*. 2013; Hu *et al*. 2020), similar neutralization potency of S14 can be expected in infectious SARS-CoV. Further validation in animal models of infectious SARS-CoV would provide more efficacy data.

The development of conventional antibodies requires considerable time and money because they usually require a mammalian expression system for production. In contrast, nanobodies can be easily expressed in microbial systems, such as bacteria, yeasts, and fungi (Mir *et al*. 2020). Frenken *et al.* reported that some nanobodies could be secreted by *S. cerevisiae* at levels over 100 mg/L in shake flask cultures (Frenken *et al*. 2000). In addition, nanobodies are thermostable. For example, nanobodies maintained full binding capacities after one week at 37 °C (Arbabi Ghahroudi *et al*. 1997). This may greatly facilitate storage and transportation for clinical applications without the requirement of strict cryopreservation. The first approved caplacizumab (Scully *et al*. 2019), a therapeutic nanobody for acquired thrombotic thrombocytopenic purpura (aTTP), suggests an extensive application prospect for nanobodies. The promising neutralization profile of S14 described here warrants further development as a therapeutic nanobody against SARS-CoV.

In summary, we identified and characterized a specific nanobody against SARS-CoV with high affinity. The *in vitro* data showed that S14 can be further tested in live virus and animal studies with some refinement of S14 to determine whether it can be used as prophylaxis or treatment of SARS, given that our experiment is no longer affected by COVID-19. In addition, similar strategies may be applied to other viruses, such as SARS-CoV-2.
